# Learning and Consolidation as Re-representation: Revising the Meaning of Memory

**DOI:** 10.3389/fpsyg.2019.00802

**Published:** 2019-04-30

**Authors:** Geraint A. Wiggins, Abdelrahman Sanjekdar

**Affiliations:** ^1^Computational Creativity Lab, AI Lab, Vrije Universiteit Brussel, Brussels, Belgium; ^2^School of Electronic Engineering and Computer Science, Queen Mary University of London, London, United Kingdom

**Keywords:** re-representation, memory consolidation, meaning construction, creativity, information dynamics

## Abstract

In this Hypothesis and Theory paper, we consider the problem of learning deeply structured knowledge representations in the absence of predefined ontologies, and in the context of long-term learning. In particular, we consider this process as a sequence of re-representation steps, of various kinds. The Information Dynamics of Thinking theory (IDyOT) admits such learning, and provides a hypothetical mechanism for the human-like construction of hierarchical memory, with the provision of symbols constructed by the system that embodies the theory. The combination of long-term learning and meaning construction in terms of symbols grounded in perceptual experience entails that the system, like a human, be capable of memory consolidation, to manage the complex and inconsistent structures that can result from learning of information that becomes more complete over time. Such consolidation changes memory structures, and thus changes their meaning. Therefore, memory consolidation entails re-representation, while re-representation entails changes of meaning. Ultimately, the theory proposes that the processes of learning and consolidation should be considered as repeated re-representation of what is learned.

## 1. Introduction: Representation and Re-Representation

This paper is about representation and re-representation, from the perspective of computational modeling of cognitive process.

The word “representation” is problematic in the interdisciplinary context of this article, because its meaning to computer scientists differs from its meaning to psychologists. The difference will bear strongly on the explanatory nature of the model proposed. In both fields, a representation (cognitive or computational) is a description of a thing, concept or state of affairs, respectively either in the memory of a computer or in the memory of a brain. But in computer science, the term also refers to the scheme or language, formal or otherwise, used to express that description—for example, a consistent set of objects and predicates capable of describing some (possibly unbounded) set of things is termed “a representation,” even before anyone has attempted to describe anything specific using it, in the sense of “a representation system.” In computer science and artificial intelligence, also, there is an element to the representation of knowledge which is usually referred to as “semantics.” This is strongly related to the idea of “semantic memory” in psychology and cognitive science, but different: the semantics is a way of writing down the meanings expressed using the representation (scheme) in such a way as to formally and consistently be able to compute with them. Mathematical logics are the most common schemes in which to express such semantics, and the representation schemes themselves are often derived from the same source (Brachman and Levesque, 1985, 1992).

The hypothetical process described here is intended to capture the process of formation of cognitive affordances that correspond, in a brain, with representation schemes (as above) in a computer. Having done so, it is then possible to build mathematical structures using such schemes which correspond with representations (in the conventional psychological sense) in brains. However, it should be noted, in comparison with, for example, Hawkins (2004) and George and Hawkins (2009) that the attempt here is not to model brain structure: functionality is modeled, but neurophysiology is not.

This is important not only because it bears upon the core contribution of the paper, but also because it entails questions about the meaning of the word “re-representation.” In the current paper, every sub-process, including the construction of representation schemes, may be viewed as re-representation, either of representational affordances, or of representations themselves, or, more commonly, both at the same time. In other words, the process of understanding the world is cast as a process of successive, stepwise re-representation. Perhaps the most unusual aspect of the theory, at least from the perspective of machine learning, is its emphasis on memory consolidation and creativity: the overarching principle is that cognitive representations enable creativity, and that re-representing them during consolidation can be creativity in its own right, and/or that re-representing can enable new creativity where previously none was possible. Evidence for this effect in humans is given, for example, by Lewis et al. (2018).

In the following sections, we first introduce the methodology behind the work. Then we introduce the background to the theory, which falls into the category of predictive cognition models (Clark, 2013). We then present the formalism used to describe memory representation and re-representation, showing where creativity is afforded. We give examples of tests which might be applied to this hypothetical model, falsifying it or supporting it in terms of the corresponding human behaviors, and we introduce some very preliminary evidence supporting the approach. Finally, we discuss the consequences of the theory for creativity in humans and machines.

## 2. Methodology: Computational Modeling for Hypothesis Development and Testing

The methodology followed in the current work is as follows. Based on a broad range of interdisciplinary background (Wiggins, 2018), and attempting to answer specific questions raised by the work of Pearce (2005), summarized below, a quantitative model is constructed, based on rigorous hypothetical cognitive principles. That model is then implemented as a computer program, which makes predictions of human behavior. Some predictions may turn out to be unexpected, and it is these that are selected for testing by comparison with human behavior (Honing, 2006). Thus, can the model be falsified, or more likely modified into a new hypothesis, presenting a properly scientific trajectory of theory development (Popper, 1959; Lakatos, 1978). Further detail of the methodology used here is given by Wiggins (2011, 2018).

The current paper contributes specifically to the trajectory of the work by opening the descriptive^1^ “black box” of the PPM* algorithm (Bell et al., 1990), used in statistical language processing, and by Pearce (2005, 2018) to allow his IDyOM^2^ system to make predictions about musical melody from statistical models. It builds on earlier papers (Wiggins and Forth, 2015; Forth et al., 2016; van der Velde et al., 2017) to continue the theoretical exposition of a hypothetical cognitive mechanism to replace PPM* as a more explanatory^3^ cognitive model.

## 3. Background: The Information Dynamics of Thinking

### 3.1. Context: Information Dynamics in Cognition

#### 3.1.1. Predictive Models of Cognition

The work is placed in a context of predictive models of cognition, as eloquently espoused by Clark (2013), and implemented over musical data using extended Markov models (Conklin and Witten, 1995; Pearce, 2005, 2018; Pearce et al., 2005; Whorley, 2013; Whorley et al., 2013; Hedges and Wiggins, 2016; Hedges, 2017). However, the core models used in that series of research are not restricted to music (Wiggins, 2012), and nor is the current one. Wiggins (2018, §2) explains why music was a good starting point for this research: music has a rather special status as a cognitive phenomenon, and affords an excellent space in which to explore ideas. Other on-going work in the area relates to the idea of “free energy” (Friston, 2010) and intrinsic motivation (Schmidhuber, 2010).

Before proceeding, it is worth noting two further potential lexical confusions.

First, the word “prediction”. The cognitive model presented here is predictive in two senses. Firstly, it simulates human predictions of what will be sensed next in the world in which it works: it simulates cognitive function that manages information received from the world by predicting what comes next, so as to make processing more efficient (Clark, 2013; Wiggins, 2018). Secondly, the model can be used to make scientific predictions about the human predictive behavior that it simulates: that is, it can predict what humans will predict when presented with a certain sequence of sensory inputs in context of a learned model.

Second, the word “model.” There are two places in this work where “model” can be reasonably used, corresponding with the two uses of “prediction.” The first is the whole theory: it is a model that simulates human perceptual predictive processing. The second is the “model” that the system builds of its own experience, which is intended to simulate human memory.

#### 3.1.2. What Is Dynamic About Information?

Information is not the same as data. Information, unlike data, is relative to a receiver, and relative to the receiver's knowledge of the transmitter(s). An extreme example is this: if Gillian and George have both read “War and Peace,” then Gillian can transmit the ideas therein to George with just those three words. If George has not read the book, it will take her somewhat longer, and many more words will be needed. Thus, shared knowledge improves the efficiency of transmission. It follows from this that as either George or Gillian learns new things, the information in a given transmission between them can change.

Shannon (1948) quantified, in the context of transmission of data along wires, how communication could be made more efficient by shared knowledge. Shannon measured information in binary integers (bits): one bit is the smallest possible unit of information. The aim of efficient communication, therefore, is to use the smallest number of bits necessary to transmit a given message. Shannon's key insight was that the entropy, *H*, associated with a transmitted symbol, *s*, drawn from an alphabet *A*, can be estimated from its likelihood in context:

(1) $$\begin{array}{l} H\left(s\right)=-\underset{s\in A}{\sum }p_{s}\log_{2}p_{s}, \end{array}$$

where *p*_*s*_ is the probability of *s* appearing after the time point in question. MacKay (2003) points out that this formula can be adapted to estimate the information content, *h*, of the actual symbol transmitted, at the time point in question:

(2) $$\begin{array}{l} h\left(s\right)=-\log_{2}p_{s}. \end{array}$$

Explicit in these formulae is that information content and entropy of a sequence of symbols is context-dependent: the existence of the prior distributions from which *p*_*s*_ is drawn expresses the effect of the context. In information dynamics models (and, we and other similar researchers claim, in predictive cognition), this distribution changes with time, each received symbol updating the model and its prediction as time proceeds. Thus, given a higher-order statistical model of a language, it is possible to compute a distribution over the words used therein, not just over the alphabet (or lexicon), but also, dynamically, over sequences in the language. Thus, at any point between two words in a sentence, we can compute a distribution expressing what is more or less expected next (by a listener who has not yet heard the next word) in terms of relative probabilities. Ungrammatical continuations will be improbable; everyday clichés will be likely; other continuations will be in between.

Thus, entropy need not be calculated from the static (zeroth-order) statistics of a model, but from dynamic distributions, changing in response to context. It is the basic contention of this kind of modeling that changes in information measures are sensed by the brain and used in its basic operation, and also available to qualia as experienced sensation (Huron, 2006).

#### 3.1.3. Information-Dynamic Measures Predict Observable Cognitive Phenomena

Models that measure the dynamics of information have been shown to predict syntactic boundaries in language (Sproat et al., 1994) and music (Assayag and Dubnov, 2004; Pearce et al., 2010b). Information content (Equation 2) predicts unexpectedness in music (Pearce and Wiggins, 2006; Pearce et al., 2010a), and measurable electrical activity in the centro-parietal region (Pearce et al., 2010a). Entropy (Equation 1) seems to predict uncertainty in music (Hansen and Pearce, 2014). The same measures seem to predict some emotional responses to music (Dubnov et al., 2006; Egermann et al., 2013). All this evidence points to one clear underlying principle: brains seem to be sensitive on several levels to information dynamics, in these two particular senses (Huron, 2006). Wiggins et al. (2015) suggest how these properties may be implicated in the evolution of creative behavior.

In the current paper, we elaborate a hypothetical process by which both representational affordances and the representations they afford, built using hierarchical information-dynamic chunking (Sproat et al., 1994; Wiggins and Forth, 2015), may, *post-hoc*, be revised and adapted to be, in a certain specific sense, better. In doing so, the meanings that the representations represent may change. In other words, the hypothesis proposes an explanation of how re-representation changes memory, and how changes in memory may induce changes in meaning. Changing meaning entails changing interpretations and perhaps understanding, and, given a mechanism to detect such change, such as a Global Workspace framework (Baars, 1988), we can relate the process of change, and the process of detection, respectively, to incubation and inspiration, in the creativity theory of Wallas (1926).

#### 3.1.4. Optimisation: Information-Efficient Representations Are Desirable Representations

A key principle in the modeling proposed here is that of information efficiency. The most information-efficient representational affordances are those which represent a given set of data using the fewest bits possible, in terms of Shannon's equations. It is easy to see, intuitively and mathematically (Equation 1) that the number of bits required to represent a symbol increases logarithmically with the number of symbols in an alphabet. Information efficiency will be an important heuristic in the following exposition. Specifically, the heuristic will be the ability of the model to predict the data from which it was learned. This is commonly applied in machine learning as cross-validation, in which parts of the data are held out, and tested against models based on the rest of the data, to verify that a particular technique is in some sense capturing the structure of the data; in statistical models, this is implemented as cross-entropy: the mean number of bits per symbol required to represent the data. Here, as explained in detail by Pearce and Wiggins (2012) the aim will be for the model to predict its own data as accurately as possible under re-representation, while keeping its mean information content, $\bar{h}$, as low as possible. Thus, the fewest possible bits are used to store the data in a useful form Wiggins et al. (2015) argue that this heuristic corresponds with an evolutionary pressure based on the biological expense of evolving, maintaining and using nervous tissue: more information-efficient is less expensive.

It is worth adding a caveat here. The aim is not simply to reduce $\bar{h}$. It is to find the most information-efficient model of the data that still allows statistically appropriate distinctions between percepts to be made. Reduction of $\bar{h}$ is the measure of success, not the goal in itself.

## 4. IDyOT Memory: Sequence and Meaning in a Self-Defining Symbolic System

### 4.1. Overview of the Information Dynamics of Thinking

The theory discussed here, the Information Dynamics of Thinking (IDyOT: Wiggins, 2018), is a statistical learning model. However, it differs from most statistical learning models in that it has related but distinct representations of sequence in time (time is itself a distinguished dimension) and of semantics, and that it is deeply hierarchical. IDyOT maintains both sequential and semantic memory, the latter being derived from the former according to particular mathematical principles. Both representations and representational affordances are constructed from sequential input, using information-theoretic measures. This process is described in outline by Wiggins (2018, p. 7.2), and summarized in the current section.

The IDyOT hypothesis enjoys the following desirable features. Some existing theories (Vernon, 2014) share some of these, but none shares all of them (Wiggins, 2018).

- It is entirely bottom-up: no assumptions about innate domain-specific properties.
- It is realistically situated in time: it learns consequence by example, so all rules it uses are learned.
- It maintains distinct sequential and semantic memories, like humans.
- It learns its memory representations bottom-up, independently of domain.
- It explicates multiple cognitive phenomena, emergent from three regular underlying mechanisms.
- It explicates semantic composition, problematic in many theories, in terms of simple association coupled with hierarchical abstraction and memory consolidation.
- It is driven by one uniform heuristic: information-efficiency. This gives it an evolutionary raison d'être, but also drives the way it discovers structure in data.

**Figure 1:**
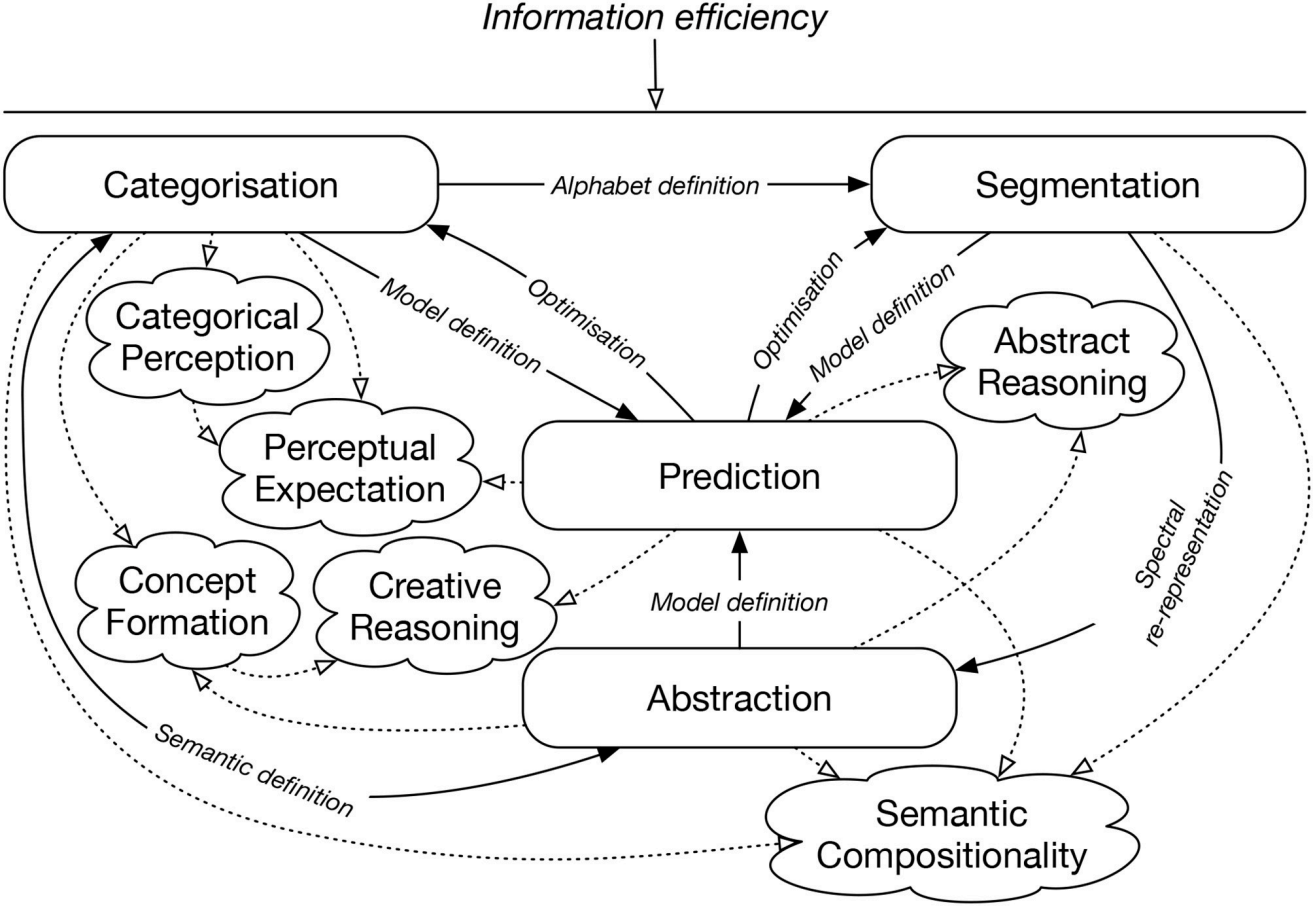
Overview of IDyOT components and operation cycle. Rounded boxes are the main IDyOT processes. Cloud shapes represent the phenomena explicated. Arrows are labeled with the operations that connect the main processes. The whole is guided by the information efficiency criterion.

These properties mean that IDyOT is not simply programmed to copy observed human behavior. Instead, it is programmed to do things that the hypothesis claims *cause* human behavior. This claim can be tested by building IDyOT and comparing its observable behavior with equivalent human behavior. We believe that IDyOT is the first theory to explicate all these components in one uniform cognitive architecture. It does so using just four inter-related mechanisms: categorization; segmentation; abstraction; and prediction. Figure 1 illustrates the overall structure of the system in terms of components and functions. It is the aim of this paper to explain these functions in detail, and so show how their combined operation enables the extraction of meaning from data, in a process of successive re-representation.

It is also appropriate to mention that IDyOT theory currently explicitly excludes a theory of forgetting, except in as far as it may be caused by consolidation (described before). This is deferred for future work.

**Figure 2:**
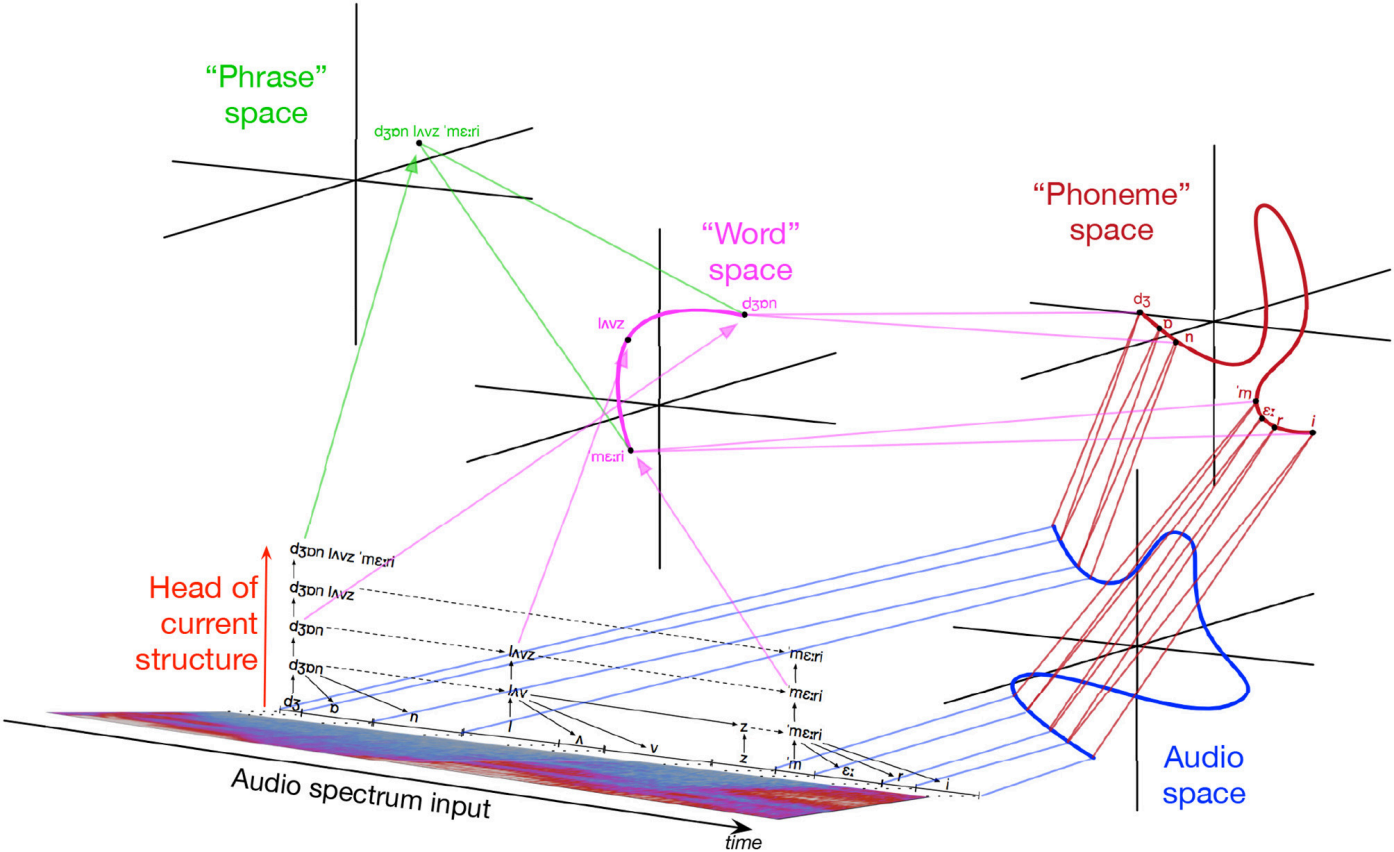
Illustration of IDyOT memory generated by speech input “John loves Mary.” Reproduced from Wiggins (2018, Figure 2) under CC-BY license.

Figure 2 illustrates IDyOT memory hypothetically generated by the speech fragment, “John loves Mary.” Detail of “loves” is omitted to avoid clutter. The audio spectrum is chunked into phonemes, then morphemes, then words, presented here in idealized form: reality is messier. As chunking proceeds, new layers are built above lower ones. As each level grows, it is chunked, building a structural representation of the input: the result is shown in black. The colored trajectories, corresponding with sequences, are in different conceptual spaces (Gärdenfors, 2000), one for each layer. Each conceptual space is a spectral representation of the layer below, so that sequences of different lengths may be compared as points in metric space. Symbols in sequential memory correspond with regions in spaces, so any symbol can be traced back to its origin in perception by following down the memory layers. In a fuller example, parallel sequences of other sensory information relating to the situation would allow associations to be developed between multiple dimensions. Wiggins (2018) shows how the combination of associative memory, hierarchical abstraction, and retrospective memory consolidation in IDyOT gives rise to the cognitively important operation of semantic composition, where meanings are combined to produce new meanings.

In this paper, we use examples that are based on discrete symbols, but if a cognitive architecture such as IDyOT is to be realistic, it must deal with continuous inputs from the world (Wiggins, 2018). It is worth mentioning, therefore, that the process of transduction, that is, of transferring sensory information from outside to inside IDyOT is included in the process of re-representation. The system works bottom-up, with a fine-grained discrete internal representation of the continuous input signal, which is progressively abstracted into larger scale symbols. Wiggins (2018) and van der Velde et al. (2017) explain this in detail.

In the next section, the processes of memory formation in IDyOT are formalized, as follows. Input sequences at the lowest level of representation, directly transduced from (possibly multidimensional) input signals, are segmented using boundary entropy. Each segment may equivalently be represented as a trajectory in a geometrical space of dimension and scale appropriate to this particular input type; this space is the ground semantic space for the modality in question. As the sequence is so described, a superordinate semantic layer is constructed, from vectors determined by spectral (e.g., Fourier) transforms of the multidimensional sequences at the subordinate layer. The representations are spectral because this mathematical approach allows comparison of sequences of varying lengths, each represented by a single point in the superordinate space. The geometry of the superordinate space is constructed by selection of an inner product function that induces a norm (distance) that models similarity in this particular space in such a way as to maximize information efficiency.

### 4.2. Formalization

#### 4.2.1. Introduction

The first novel contribution of this paper is a formal specification of IDyOT memory formation, which allows the subsequent specification of a hypothetical mechanism for memory consolidation. We begin with the construction of sequential memory, showing first how it gives rise to semantic memory, then to chunking, and then to hierarchical memory in turn. The key point to observe is how representational affordances (inherent in semantic spaces) are inferred from representations (derived from data by means of existing semantic affordances).

#### 4.2.2. Representation

IDyOT maintains a literal record of its sensory input. In humans, of course, this is not the case; however, such a record can be more easily removed or ignored than it can be reconstructed, so it is included in the formal model. The key factors of the model are as follows.

Time, in terms of sequential time units with a duration, is divided into discrete moments, denoted τ below; a given τ should be thought of as marking the start of the moment. Boundaries of moments are identified using the segmentation process described below. Real-time elements of IDyOT are discussed by Forth et al. (2016), and is not covered here.

Sensory input, divided into moments, is then viewed as a single multidimensional entity, the coincidence of values in its dimensions being potential evidence of correlation. Dimension number is denoted δ.

IDyOT memory is hierarchical, and the method for constructing the hierarchy is one of the novelties of the theory. Hierarchical abstraction (for the levels capture progressive representational abstraction in a particular sense: Wiggins and Forth, 2015) is denoted α. Abstraction will entail construction of new dimensions, so where there is an α parameter, there is a matching δ.

Given these parameters, a symbol in IDyOT's sequential memory is denoted $S_{\tau }^{\delta ,\alpha }$. The alphabet, *A*, from which these symbols drawn is strictly partitioned on δ and α, denoted *A*^δ,α^. Furthermore, *A* is dynamic, and it is this capacity for change that admits consolidation and re-representation. In the following discussion, a set of dimensions representing audio input (specifically, speech), will be used for illustration. The dimension parameter, δ, is used here to identify a fresh sequence of symbols at any level, α: thus, an input sequence has a δ value, and so does a new sequence drawn from a new alphabet, created as described below. The level α = 0 dimensions used to represent an audio signal (in the illustration a spectral transform, such as Fourier, simulating the effect of the Organ of Corti) are, in the terms of Gärdenfors (2000), an integral set of dimensions. That is to say, the information contained in each dimension has meaning only in context of the other dimensions in the set. Here, they define a conceptual space of sound (Gärdenfors, 2000). As such, we can, for the purposes of illustration, think of them as one. In this illustration, too, we assume that the difficult problem of source separation has been solved^4^: the notional input is a clean representation of an isolated voice.

**Figure 3:**
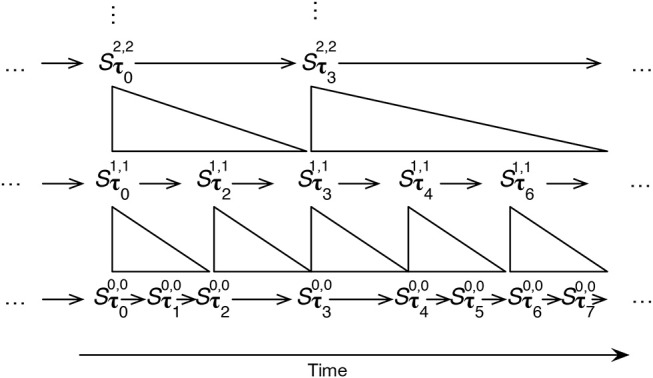
A schematic representation of IDyOT sequential memory for a single dimension, the dimension parameter being omitted. τ_*i*_ denote the *i*th moment in the time as it is memorized, and time proceeds from left to right. The arrows within the layers denote sequence in time. The structures between the layers denote the segmentation relationship between the layers, described below. Note that moments at different layers can have different lengths.

Given this notation, and simplifying to one δ dimension, IDyOT sequential memory, may be thought of as being like the layered structure shown in Figure 3.

#### 4.2.3. Prediction in Sequence

We now formalize the idea of prediction. A simple bigram model (Manning and Schütze, 1999, Ch. 9), *M*^δ,α^, can be constructed over alphabet *A*^δ,α^ from any (integral set of) dimension(s), δ, and any layer, α, for a structure such as that in Figure 3. The contiguity requirement implicit in a standard bigram model means that a simple notation will be useful: $\tau_{+i}^{\delta ,\alpha }$ denotes the moment of the *i*th symbol following τ in dimension δ, level α; similarly, $\tau_{-i}^{\delta ,\alpha }$ denotes^5^ the moment of the *i*th symbol preceding τ^δ, α^. Thus, the model *M*^δ,α^ associates each contiguous pair of symbols in the sequence with the likelihood that pair of symbols appears contiguously, counted from the memory^6^: $\langle S_{\tau }^{\delta ,\alpha },S_{\tau_{+1}}^{\delta ,\alpha },p_{\tau_{+1}}^{\delta ,\alpha }\rangle \in A^{\delta ,\alpha }\times A^{\delta ,\alpha }\times \left[0,1\right]$. Given *M*^δ,α^, we can compute a distribution $D_{\tau }^{\delta ,\alpha }$ over *A*^δ,α^ at any moment τ, expressing the expectation inferred from the model, of what is to come at moment τ_+1_. Then, using Equation 1, we can compute the Shannon entropy on dimension δ, at level α during moment τ:

(3) $$\begin{array}{l} H\left(\delta ,\alpha ,\tau \right)=-\underset{\langle S_{\tau }^{\delta ,\alpha },S_{\tau_{+1}}^{\delta ,\alpha },p_{\tau_{+1}}^{\delta ,\alpha }\rangle \in M^{\delta ,\alpha }}{\sum }p_{\tau_{+1}}^{\delta ,\alpha }\log_{2}p_{\tau_{+1}}^{\delta ,\alpha }, \end{array}$$

noting the unusual notation, which expresses the values in terms of particular points in IDyOT memory, rather than of the alphabet *A*^δ,α^, as is more common. Similarly, using Equation 2 the information content of the symbol at moment τ in level α is:

(4) $$\begin{array}{l} h\left(\delta ,\alpha ,\tau \right)=-\log_{2}p_{\tau }^{\delta ,\alpha }, \end{array}$$

where

$$\langle S_{\tau_{-1}}^{\delta ,\alpha },S_{\tau }^{\delta ,\alpha },p_{\tau }^{\delta ,\alpha }\rangle \in M.$$

An advantage of this notation is that it makes very clear a difference in meaning between the Mackay's two applications of Shannon's idea (MacKay, 2003): *H* is a quantity which in some sense refers to the next moment, because it is a function of the current one; *h*, however, is a value associated with the current moment as it appears in context.

The within-layer bigram model afforded here is substantially simpler than that used in successful modeling of musical syntax by Conklin and Witten (1995) and of music perception by Pearce and Wiggins (2006). This extra complexity of the earlier models, inside the black box of the PPM* algorithm (Bell et al., 1990) is displaced into interactions between the abstraction layers, α, in IDyOT, and the detail of its operation is not central to the argument presented here. Suffice it to say that predictions at any given layer may be modulated by predictions in neighboring levels, in the way similar to that in which distributions from different representations may modulate each other in Conklin's viewpoint system (Conklin and Witten, 1995; Pearce et al., 2005).

Given these mathematical tools, the next section explains the construction of semantic memory in IDyOT.

### 4.3. Semantic Memory in IDyOT

#### 4.3.1. Semantic Space

Wiggins (2018) section 7.2 introduces IDyOT semantic memory. Semantic memory, as in most cognitive architectures (Vernon, 2014), is memory for meanings, abstracted away from the events stored in the sequential memory. It may be thought of as simulating the feeling of meaning, as opposed to memory of specific events. It is semantic memory that allows IDyOT to represent similarity and difference between percepts and objects, and thus become a reasoning entity (Gärdenfors, 2000). IDyOT semantic memory is constructed as follows.

At level α = 0, the semantic memory is the direct representation of the percept, as measured in the input: it is, at this level, its own meaning. For example, in the case of audio, the input is in the form of short-term spectra, one for each moment, representing input to the lowest level auditory cortex from the organ of Corti. These spectra, and other quasi-continuous perceptual inputs, may be viewed as vectors in a Hilbert space (Hilbert, 1992), denoted in general *V*^δ,α^, and specifically *V*^δ,0^, here. Such a space is composed of three elements: a set of vectors, an inner product function over that set, and a norm, which is analogous to a distance in physical space, within *V*^δ,α^. The inner product defines the geometry of the space; the norm is induced from the inner product. So, in general,

(5) $$\begin{array}{l} V^{\delta ,\alpha }=\langle S_{,}^{\alpha }\times_{i},{∥.∥}_{i}\rangle \end{array}$$

where *i* is an index that admits the existence of multiple vector spaces, with different inner product functions, over the same set of vectors, thought of as a library of possibilities. In this way, IDyOT can support multiple views^7^ over its raw data. For example, musical pitch may be considered, simultaneously, as a line of pitch height, a circular scale position, and/or a spiral that combines the two, and any of these may be considered alongside intensity. The precise nature of × _*i*_ depends on various factors, and is a matter for future study in IDyOT. Clues already exist: (Kemp and Tenenbaum, 2008) propose that certain mathematical structures are preferred within human brains; while multiple attempts exist in music signal processing^8^ to attempt to infer similarity of various kinds by inferring the necessary inner product from empirically derived partial norms and sets of data points. Wiggins (2018) section 7.25 explains why the mathematical power of Hilbert space representations is appropriate here.

It is important to understand that the semantic spaces are, at this low level, not the same as the denotational semantics of linguistics. Rather, they capture the grounding of meaning in sensation. Recall, also, that in the general case, input to IDyOT will be from multiple sensors, so that statistical associations between sensory inputs, moment by moment, may also be calculated. It follows that the meaning that these points represent may not have a specific name, but instead a complex description related to their source, such as “the color we picked in Photoshop at 5:30 p.m. yesterday.”

The key idea, here, is that each vector in the semantic space captures the sensory meaning experienced in the moment to which it corresponds, and the geometry of the space captures the similarity relation between those meanings, as follows.

#### 4.3.2. Similarity and Categorization in Semantic Space

Given at least one *V*^δ,α^, it is now possible to discuss similarity, which is defined by the norm, ∣∣.∣∣_*i*_, under the procedure described here. Gärdenfors (2000) suggests that convex regions in spaces such as these correspond with natural cognitive concepts, such as colors and sizes; the norms may be used, analogously with distance between points in a Cartesian space, to identify the centroid (or other mathematical summary) of a set of points, as a conceptual prototype. By measuring the distance from such a prototype, one might measure typicality, and eventually decide which particular points were included in, or excluded from, the concept. Gärdenfors (2000) argues convincingly that the key issue here is betweenness: that is to say, it should be possible to identify an item that is between any two others in the same category, and the function of such perception that justifies its evolution is the ability to distinguish between things.

The formation of such conceptual categories is fundamental to the operation of an IDyOT. The bigram model constructed from a set of vector pairs such as that described in the current audio example would be hopelessly sparse, because there is a very low likelihood that any two spectra would be identical. Therefore, to construct a meaningful model, *A*^δ,1^, from *A*^δ,0^, it is necessary to partition *A*^δ,0^ into equivalence classes, or regions in *V*^1^, allowing the data to be represented less sparsely, though approximately. These regions can be labeled with a representative point, such as their centroid, so that identifying them is a simple equality test. This simulates the beginning of categorical perception, and constitutes the first (very simple) example of re-representation in the model. The question, of course, is: which vectors should be in which subset?

The overall effect of this partition, whose relation is in general denoted $\Pi_{\delta_{1},\alpha }^{\delta_{2},\alpha +1}$, is to reduce the effective size of *A*^δ,1^, which in turn has the effect of reducing expected information content. To see this, observe the case of maximum entropy, when the distribution across the alphabet (of any level) is uniform, denoted Ĥ^α^. Then, probabilities decrease with increasing alphabet size, and so Ĥ^α^ increases. Thus, partitioning *A*^δ,0^ as described reduces the size of the required alphabet and moves the layer in the direction of the information-efficiency heuristic. However, the logical extreme of this transformation is to include all symbols in the same category, reducing ${\bar{h}}^{\delta ,\alpha }$ to zero, but rendering the model vacuous. This would be undesirable.

**Figure 4:**
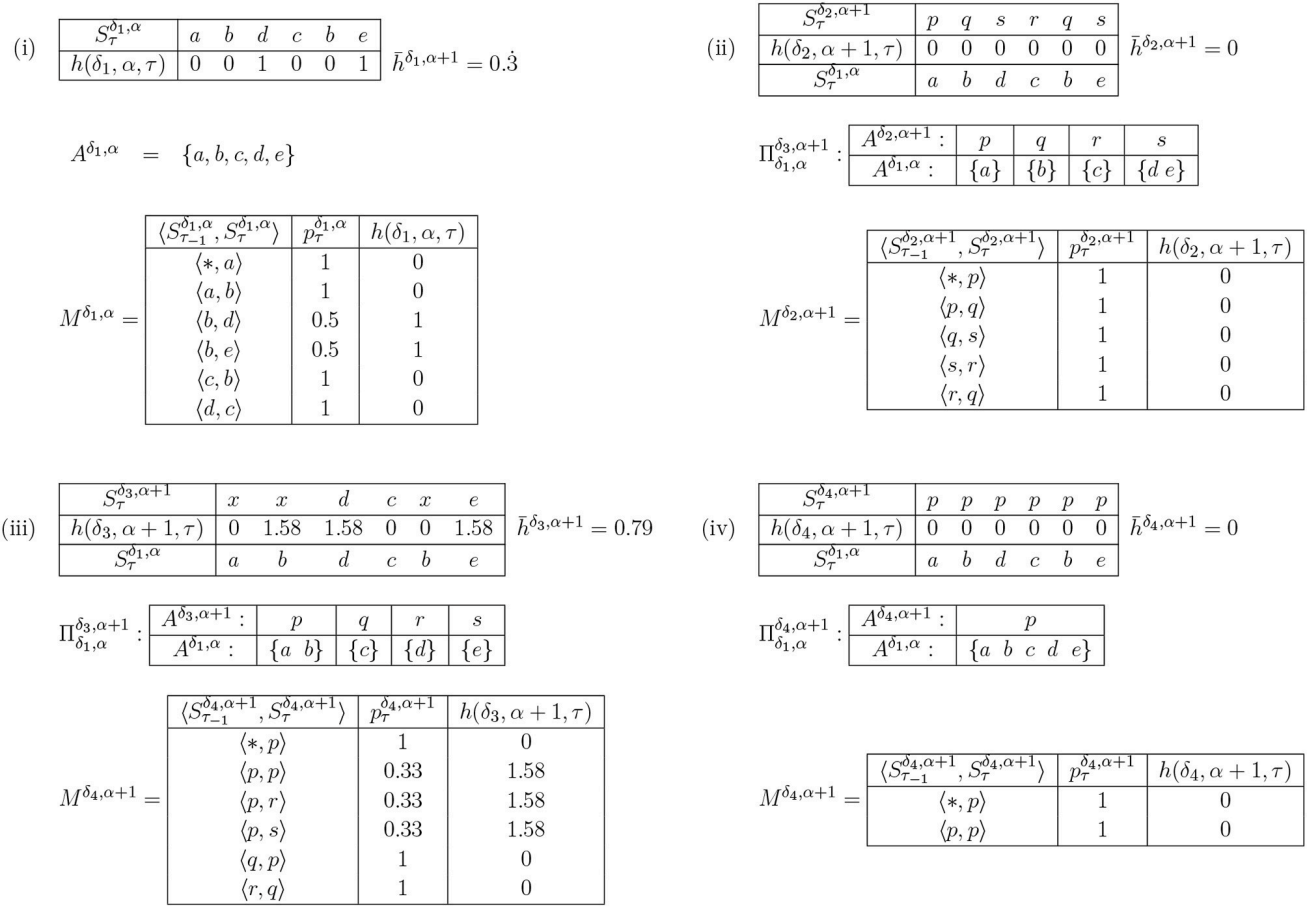
An example of ${\bar{h}}^{\delta ,\alpha }$ reduction by heuristic selection. **(i)** shows a very simple example of IDyOT memory (the assumption here is that this is the entire memory). Shown first, in **(i)**, is the memorized sequence along with the information content of each moment. This example is simplified, because the model is derived from the entire string, *post-hoc*. In a working IDyOT, the statistics would be changing as the string was read. Shown next is the alphabet, *A*^δ,α^. Finally, the bigram model, *M*^δ,α^, is shown (missing pairs of symbols have zero likelihood). It is clear, in this simple case (but also in general), that the uncertainty in the model arises from the choice following a given symbol, here a *b*; therefore, this is where an attempt should first be made to reduce the alphabet. This is shown in **(ii)**, where $\Pi_{\delta_{1},\alpha }^{\delta_{2},\alpha +1}$ is a partition of $A^{\delta_{1},\alpha }$, so that *d* and *e* are in the same partition, called *p*. In this instance, all the uncertainty is removed, and ${\bar{h}}^{\delta ,\alpha }$ for this memory is 0. A new expectation is created for the following symbol, *c*, which was not available before the categorization, and this is the first glimmer (however small) of creativity caused by re-representation. However, it is possible that this transformation might be vetoed by appeal to the convexity of the resulting region in $V^{\delta_{2},\alpha +1}$; if so, case **(ii)** is discarded. Case **(iii)** shows, for comparison, the effect of categorizing a random pair of symbols, *a* and *b*, earlier in the process. Clearly, here, ${\bar{h}}^{\delta ,\alpha }$ increases, and it is easy to see why: the change introduces new uncertainty into *M*^α+1^ which directly affects ${\bar{h}}^{\delta ,\alpha }$ of the memory, and therefore this potential categorization should be rejected. For completeness, case **(iv)** shows the case where all the symbols are categorized into one partition. This should never arise in practice, under the above procedure, because it is highly unlikely that ${\bar{h}}^{\delta =,\alpha 0}$ would be achievable for a non-trivial example.

The solution to this issue is to reduce the *A*^δ,α+1^ incrementally, and only when doing so corresponds with appropriate clustering of the latest symbol in at least one *V*^δ,α+1^. To put this another way: a new symbol may be merged with an existing one only when they both fit naturally into a semantic category, and in line with this, we term the process categorization. Categorizing a number of symbols into one Gärdenforsian concept must be licensed both by a decrease in ${\bar{h}}^{\delta ,\alpha }$, and also the existence of at least one × _*i*_ under whose norm the two symbols may be considered in the same category. The primary candidates for categorization are pairs of symbols which have at least one predecessor in common, and which therefore have *h*(δ, α, τ) > 0, though all combinations with the newest symbol must be considered (Figure 4): an appropriate heuristic measure would be to order candidates by decreasing *h*(δ, α, τ). Given such a pair, one or more corresponding *V*^δ,α^s must be selected, according to the following rules. Candidates may be ruled out using Gärdenfors' convexity criterion: they cannot be considered for categorization if there exists a member of the *A*^δ,α^, outside the candidate set, which is closer to the centroid of the resulting candidate set than a convex hull around the candidate points: the extent of that hull beyond the points themselves is defined by a parameter, ρ, which determines how willing the IDyOT is to reject possible categorizations. The effect of this is that two candidates cannot be categorized together if doing so would create a non-convex region in the radius of ρ. The candidate sets are considered sequentially, ordered by total mean information content, highest first. If any such *V*^α+1^ can be found, corresponding new dimensions at level α + 1 are created. If none is found, then the categorization fails. The heuristic selection is illustrated in Figure 4.

Since the relation between two layers in categorization is essentially the subset relation, in the event that multiple categorization layers are created, without segmentation layers (explained in the next section) between, they may be compressed into one single layer, thus simplifying computational implementations. However, it is convenient to keep the full construction history visible in the mathematical statement of the theory.

### 4.4. Chunking/Segmentation by Boundary Entropy

Given a layer of categorized symbols as generated above, it is possible to segment the sequence of a given layer by means of boundary entropy. The rationale for this is as follows. In order to cope with the vast amount of information available in the world, it is necessary for a perceiving organism to compress it. A good method of compression is to recognize repeated structures, ideally where approximate repetition is included. If the sequential inputs of IDyOT contain repeated structures, then, statistically, as a repeated segment is processed, the symbols in it become progressively less and less unlikely; in Shannon terms, they convey progressively less information, and they become more and more certain. However, when the end of a repetition is reached, the next symbol becomes less predictable. Shannon entropy and information content can be used to segment both language and music (Sproat et al., 1994; Wiggins, 2010; Pearce et al., 2010b) in ways that correspond with analyses by linguists and musicologists, respectively. To do this, one examines the time-variant signal produced by the Shannon formulae, and identifies sharp increases in entropy or information content (Pearce et al., 2010b), with respect to local context (Pearce et al., 2010b) or an absolute value (Wiggins, 2010, 2012). The exact specification of the detector required is a matter for further empirical study, since there is currently no clear winning candidate in the literature; here, it is denoted by $\Delta_{\tau }^{\delta ,\alpha }$. It yields a Boolean value (true, iff τ is the first moment in a new segment). The definition we will use for illustration here is as follows; it combines entropy and information content:

$$\Delta_{\tau }^{\delta ,\alpha }=\{\begin{array}{cc} \text{true} & \text{if}H(\delta ,\alpha ,\tau_{-1})<H(\delta ,\alpha ,\tau ),\\ \text{true} & \text{if}H(\delta ,\alpha ,\tau_{-1})\geq H(\delta ,\alpha ,\tau )\wedge h(\delta ,\alpha ,\tau )<h(\delta ,\alpha ,\tau_{+1}),\\ \text{false} & \text{otherwise.} \end{array}$$

**Figure 5:**
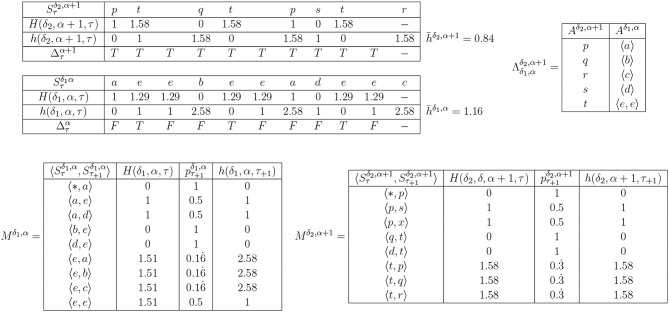
An example of IDyOT seqmentation. For illustrative clarity, as in Figure 4, *M*^δ,α^ is constructed statically *post hoc*, and not incrementally. The figure shows levels α and α+1 for an example sequence similar to that in Figure 3. *H* is not calculable for a symbol which has not been encountered as the first element of a bigram, so no value can be given for the last symbol. Note that the sequence “*ee*” arises more than once: this is enough to suggest that it might be treated as a segment at a higher level. Note also that ${\bar{h}}^{\delta ,\alpha }$ of layer α+1 is lower that of layer α, and that layer α is impervious to more grouping under the current definition of $\Delta_{\tau }^{\alpha }$, so the process stops.

Given $\Delta_{\tau }^{\delta ,\alpha }$, as shown in Figure 5, we can detect candidate boundaries, either along a complete layer, or incrementally, as the layer is assembled. The latter is the approach taken in IDyOT: the aim at this point is to model ongoing, waking, non-conscious perception. Necessarily, and especially at the beginning of an IDyOT's memory, the incremental nature of the process will result in sub-optimal learned model, because the predictions of the model are not informed of the future. However, this is a price worth paying in comparison to the computational cost of constantly revising the memory as it is updated, or frequent repeated stopping of the learning process in order to consolidate, using the procedure proposed below. The former would either require excessive expenditure of neural capacity or an organism that acts more slowly, making an organism vulnerable to predators. The latter would require frequent regular short periods of unconsciousness, again placing an organism in danger of predation. As such, neither is likely to be favored by evolution over maximal waking activity. Instead, the compromise experienced by modern humans is modeled^9^: rapid, continual on-line learning during waking time, followed by sustained periods of consolidation during sleep. The consolidation phase reduces the importance of the correctness of individual segmentation decisions, on the grounds that if they are incorrect, they can be corrected later.

Segmentation will, below, allow the construction of the hierarchy illustrated in Figure 3. First, however, it is necessary to consider the meaning of segments. Chella et al. (2008) address the problem of modeling sequences in conceptual space theory, in the context of robot movements^10^. The issue here is that it is necessary to identify a range of slightly different movements as essentially the same movement, when it is so, notwithstanding local variations of location and speed. Chella (2015) extends the same notion to music perception, both at the levels of pitch and timbre, and at the level of musical phrase (that is, of sequences of notes). The mechanism proposed here builds on these ideas to explain how the models are constructed. Chella's key idea is to use spectral transforms to abstract out the temporal element of sequences that may be of arbitrary length, and convert them to more abstract representations, where sequences become points in (more abstract) conceptual spaces. The resulting relation, in IDyOT theory, is called $\Lambda_{\delta_{1},\alpha }^{\delta_{2},\alpha +1}$, and it stands in correspondence with $\Pi_{\delta_{1},\alpha }^{\delta_{2},\alpha +1}$ in the sequential memory; that is, $\Pi_{\delta_{1},\alpha }^{\delta_{2},\alpha +1}$ and $\Lambda_{\delta_{1},\alpha }^{\delta_{2},\alpha +1}$ serve a corresponding purpose in the sequential and semantic memories, respectively: to link a symbol or region, respectively, at a given level with a sequence or trajectory, respectively, at the level below. It relates regions in a given $V^{\delta_{2},\alpha +1}$ with trajectories in the corresponding $V^{\delta_{1},\alpha }$, or, equivalently, points in a given $V^{\delta_{2},\alpha +1}$ with trajectories containing points in a corresponding $V^{\delta_{2},\alpha }$. By analogy with geometry, this relation is termed “subtend”, in either memory, so $A^{\delta_{2},\alpha +1}$ subtends the sequence in $A^{\delta_{1},\alpha }$ under $\Pi_{\delta_{1},\alpha }^{\delta_{2},\alpha +1}$ and $V^{\delta_{2},\alpha +1}$ subtends the trajectory in $V^{\delta_{1},\alpha }$ under $\Lambda_{\delta_{1},\alpha }^{\delta_{2},\alpha +1}$. The real-time element of this relation is handled separately from other dimensions, as explained by Forth et al. (2016); this is not relevant here.

A detailed example of IDyOT segmentation is given in Figure 5. The input sequence used is constructed to include something that could be construed as a word or some other linguistic group: the sequence “*ee*” appears three times, whereas other symbols show no regularity. The information-theoretic chunking mechanism identifies this and creates a new symbol to subtend the repeated sequence, thus reducing average information content. However, the symbol chunking shown in the figure is only part of the story. Layer α already has at least one $V^{\delta_{1},\alpha }$, which means that it is possible to construct a $V^{\delta_{2},\alpha +1}$ whose points are spectral transforms of the points corresponding with the symbols from *A*^δ,α^ constructed into (sometimes single-point) trajectories, as defined by $\Lambda_{\delta_{1},\alpha }^{\delta_{2},\alpha +1}$. The same rules apply to the construction of *V*^δ,α+1^ as in the categorization phase, above: a suitable × _*i*_ must be found. Thus, it becomes possible to link complex sequences of meanings with individual summarizing concepts, and therefore the first step to learning grammar^11^ is taken. Like categorization, segmentation is a process of re-representation, because a new level of representational affordance is generated, in which sequences of symbols at the lower levels may be compared and categorized.

### 4.5. Hierarchy Construction

Given the two re-representation processes proposed in the two foregoing sections, it is now possible to specify an algorithm for processing symbols as they appear, interleaving the processes repeatedly until neither of them can go further, and the resulting memory is irreducible. Doing so will produce a hierarchy of the kind illustrated in Figure 3. It is implicit in this process that different segments at one level of abstraction may be grouped together at a higher level, if such grouping is licensed by an appropriate semantic space. In this way, categories of similar meanings may be constructed, providing the next step toward grammar construction.

Both kinds of alphabet reduction, categorization and segmentation, may involve choices. Alternatives can be dealt with in several ways in the context of a process such as this: (1) choose a representation, based on the heuristics introduced above, and stick with it—this is called a greedy strategy in studies of search methods; (2) explicitly represent the ambiguity using multiple spaces (for categorization) or segmentations (for segmentation) in parallel at any given layer; (3) allow the system to backtrack, undo and redo decisions in the face of new evidence. Definitive answers to these choice are a matter for empirical research. However, an outcome consistent with the principle of information reduction, and therefore that proposed here, is that segmentation strategies will reduce wherever the available evidence allows, taking the greedy option, and perhaps allowing a minimum of local parallelism. Doing so means committing to answers where there is ambiguity, thus sometimes ruling out possibilities, and possibly missing preferable solutions. But to avoid this option would mean expensive use of memory, either to record which decisions were taken last, what they were, and what was their context (and so allow decisions to be unpick and redone), or to maintain multiple segmentations in parallel. While multiple segmentations are indeed possible in the formalism (because of multiple semantic spaces, δ, and any given α level), their expense should be avoided wherever possible, for the reasons already rehearsed above. Ambiguity, or rather multiplicity, of categorization, however, is an active part of cognitive construction of a world model, as demonstrated by Saffran and Griepentrog (2001), and intuitively felt by any experienced musician, in the context of musical hearing of both relative and functional pitch^12^ at the same time; in speech, examples are the ability to hear pitch, timbre and intensity as separate features of a signal. Therefore, such representational multiplicity, which admits cognitive flexibility, and the possibility of analogy and metaphor (McGregor et al., 2015) should be supported: the dimension labeling, δ, in the formalism makes this possible.

It is for these reasons, of practical compromise, that IDyOT theory includes a model of memory consolidation, viewed as a process of optimisation of the memory structure described above, so as to be self-consistent and information-efficient. This model is the subject of the next section.

## 5. Hypothesis: A Mechanism for Memory Consolidation

The process of memory consolidation in IDyOT, like the rest of IDyOT's processes, is deliberately simple, constituting an initial hypothesis as a starting point for empirical research. However, as with the rest of IDyOT's processes, because of the recursive nature of the system, simple effects can have major consequences. Here, as seen in a very small way in the example of Figure 4, they can enable creativity, and they can support the learning of sequential phenomena as complex as human language.

During a period of learning, IDyOT will operate as described above, greedily optimizing its learning. However, at some point, it must stop and consolidate, loosely simulating the point that humans must stop and rest or sleep. The aim of this consolidation is to heuristically minimize the mean information content, $\underset{\delta ,\alpha }{\sum }{\bar{h}}^{\delta ,\alpha }$, of the entire memory. For any IDyOT with the opportunity to develop over a long period, an analytical, non-heuristic approach to this would mean very substantial computation, which is not necessarily desirable in a cognitive model: the aim here is not necessarily to achieve the smallest $\underset{\delta ,\alpha }{\sum }{\bar{h}}^{\delta ,\alpha }$, but to represent the learned information in the way that predicts the world best.

In order to implement consolidation, therefore, propose a heuristic method based on information content. Overall, the aim is to locate the part of the memory that models its data the least well, and therefore has the highest information content, and adjust a minimal number of symbols in such a way that the model is better. A precursor of this idea was seen in Figure 4ii where ${\bar{h}}^{\delta ,\alpha }$ was reduced by the categorization of two symbols, which directly caused uncertainty and therefore increased information content; categorizing those symbols together (if such an act were licensed by the existence of an appropriate semantic space) removed the uncertainty, and thus reduced $\underset{\delta ,\alpha }{\sum }{\bar{h}}^{\delta ,\alpha }$.

Once off-line, or receiving no data, the processes of categorization and segmentation as described above may proceed in a non-sequential manner: there is now no requirement to function quickly, as events in the world unfold. This means that the memory may be examined thoroughly, and the sources of uncertainty and/or high information may be identified. Furthermore, it means that repairs, which may take non-trivial processing effort, may be applied. From the perspective of computational implementation, these quantities are cheap to calculate, so an efficient heuristic search may be made through the memory.

Four consolidation operations may be applied here: (i) categorization, as before, but applied in heuristic order along the entire memory; (ii) segmentation, as before, but applied in heuristic order along the entire memory; (iii) re-categorization, the converse of categorization, where it transpires that separating elements of a category into two or more sub-categories is desirable; and (iv) re-segmentation, in which existing segments are themselves split as a result of new evidence. Note that these operations may be chained so that symbols effectively move from one category to another or from one sequence to another. Each one of these may require processing at multiple levels and in multiple parts of the memory. Furthermore, because IDyOT memory is one interconnected network, each change is likely to cause further changes that will ripple upwards through it, until eventually a newly stable overall representation is reached, or until time for consolidation is exhausted. In principle, it is clear, new representations introduced as part of this process can change the meaning of symbols, because their grounding in perception can change, as can their relation to other categories in their defining *V*^δ,α^. Thus, new representations can be formed, new categories within semantic space defined, and points may be moved from one category to another.

Thus, the hypothetical algorithm proposed here is a therefore a simple loop, shown in Algorithm 1. In English: Take the symbol with the highest information content. Attempt to categorize it with others. Then attempt to segment around it. If either or both attempts succeeded, choose the resulting symbol with highest information content, and start again. If neither attempt succeeded, choose the next highest information content and start again. Repeat this process until the available time is exhausted.

**Algorithm 1:**
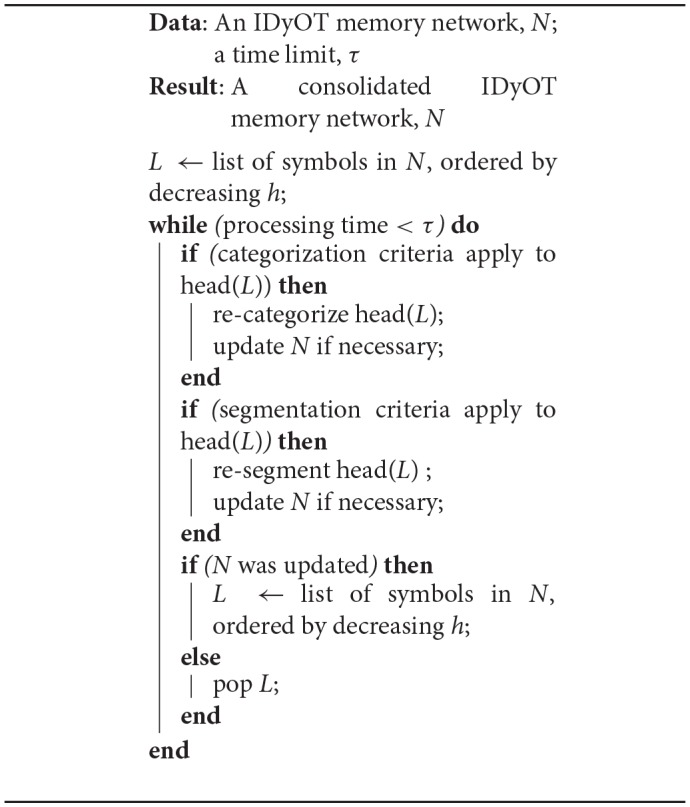
IDyOT Memory Consolidation

## 6. Testing the Hypothesis

### 6.1. Approach

For all this model may be explained in relatively simple terms, because of its iterative nature, complex, long-term consequences are difficult to predict. It is part of the methodology used here that one should build the model, run it, and identify unexpected predictions—the more unexpected the better (Honing, 2006); these predictions should then be tested against human behavior, to validate or falsify the model. However, equally, without an implementation, one should be able to predict simpler outcomes. This section gives an example of such an outcome, in word re-segmentation.

**Figure 6:**
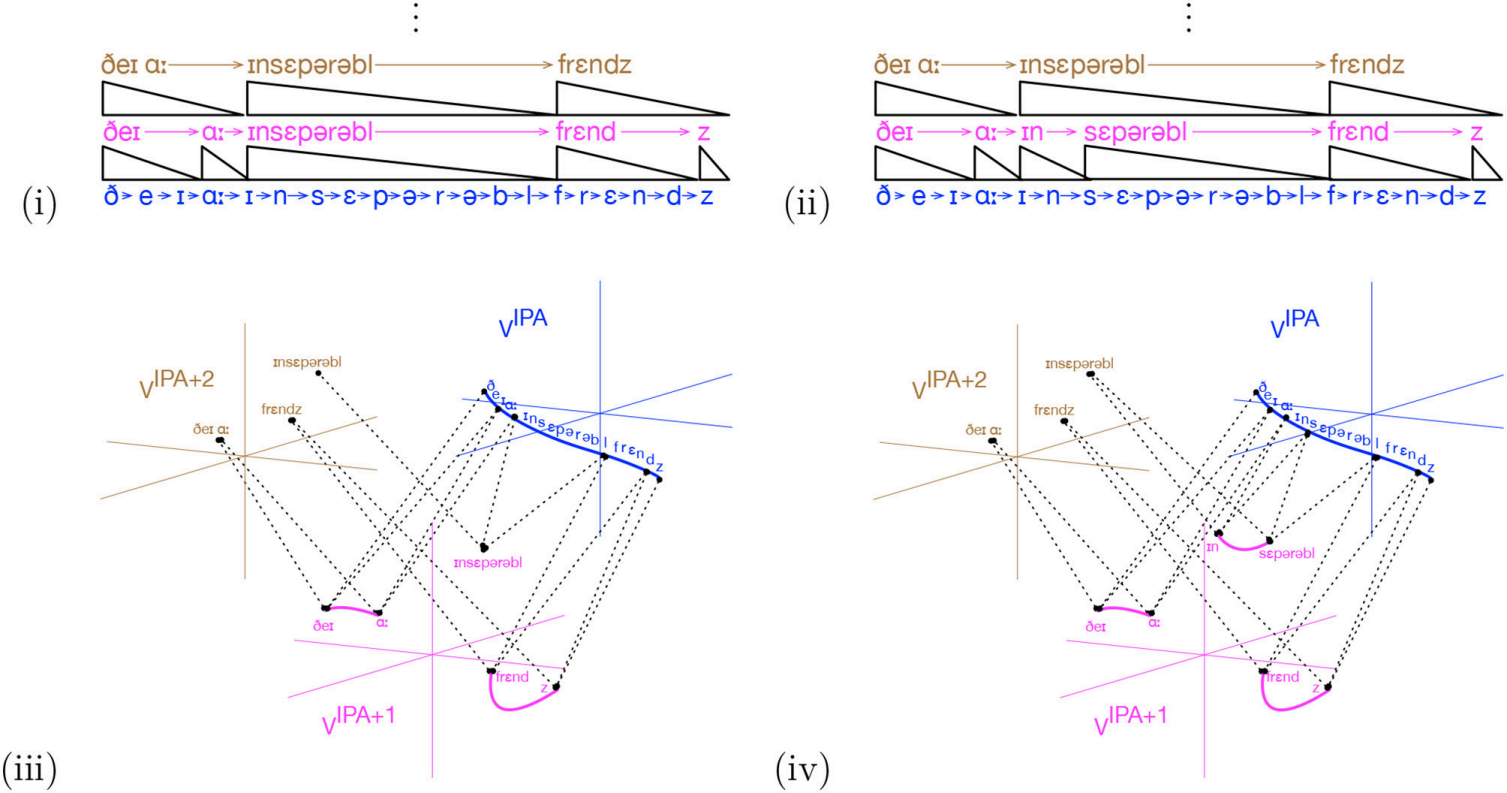
Re-segmentation of the word “inseparable” under consolidation. The initial IDyOT memory **(i)** is revised on the basis of further evidence to explicitly represent the negating prefix, yielding **(ii)**. The corresponding semantic representations are shown in **(iii,iv)**. Note that **(iv)** now affords a label to associate with the concept of logical negation.

It is a feature of human memory that the segmentation of audio streams may be revised. To see this, consider the word “inseparable.” Imagine an IDyOT that has not yet encountered the negating prefix “in” or the word “separable”, in its learning, and which is now learning the sentence, “They are inseparable friends.” for the first time. Supposing that the IDyOT is reasonably otherwise experienced^13^, it might produce a representation as shown in Figure 6i, using the International Phonetic Alphabet, *A*^IPA^, as a proxy for the categories learned for low level speech by the IDyOT. Now suppose that, over a period, the phrase “in a separable way” is encountered, along with other instances of “in.” This will change *M*^IPA^ in such a way as to make Δ^IPA^ true at the start of the first moment of “separable.” In the next consolidation phase, if there is enough time and if *h*^IPA^ is large enough, the initial input will be re-segmented is shown in Figure 6ii. In this (rather contrived) instance, there need be no further ripple effects: “inseparable" was already a segment at the higher level. The identification of the distinct “in” prefix causes a reduction in *h* for any IDyOT with knowledge of more than two uses of it: for *n* such adjectives, there are 2*n* corresponding symbols—the positive and negated versions–in the unseparated form; with the distinct prefix “in”, there are *n*+1. It also affords a new representation, as shown in Figure 6iii, iv: a label with which to associate the negation of concepts in general. What an example on paper cannot show is other information from sensory context and higher-order reasoning afforded by the higher layers of IDyOT memory that conspire to represent the meaning of the prefix “in” and the word “separable,” which, after (iv) are necessarily associated with the corresponding sections of speech. This, again, is a simple kind of creativity: while the discovery of this idea is certainly prompted by its existence in the speaker of the input language, from the perspective of the individual IDyOT, its generation is, in the terms of (Boden, 2004), P-creative.

Such behaviors are observable in humans, and can be tested using languages generated artificial grammars, to which an IDyOT can be exposed in comparison with humans. Thus, a programme of research can be designed in which memory revision and consolidation in humans is compared with that in IDyOT theory. The same method can be applied in music (e.g., Pearce and Wiggins, 2006), though it is harder to dissociate musical enculturation from human responses to pitch patterns than with artificial “word” strings.

### 6.2. Some Very Preliminary Supporting Evidence

In this section, we present some very preliminary supporting evidence that IDyOT's re-representation approach does indeed contribute useful information. The evidence comes from an implementation, which is work in progress by the second author. At time of writing, the implementation covers only the sequential aspect of IDyOT, replacing the subtlety of semantic spaces with simple equality, and it includes only the effect of lower layers in the sequence hierarchy on higher ones, and not the converse. It contains no consolidation phase. Thus, the difference between this proto-IDyOT and a simple bigram model is the stacking of successive layers' models, and the consequent passage of segmentation information up the network.

#### 6.2.1. Method

We have begun to implement IDyOT as a Java program, and our results come from the empirical application of that programme. We are considering here a well-defined data set, with clear symbols and a clear ground truth, so as to run the system in a readily verifiable context; thus can we understand its operation better. More challenging data will come later.

Our data is three novels in English: The Time Machine (H. G. Wells), Moby Dick (Herman Melville) and Pride and Prejudice (Jane Austen), drawn from the Project Gutenberg database^14^, including any surrounding material that Project Gutenberg has added. The aim of the study is to segment these books in such a way that words are correctly found, the ground truth being the original texts, and the input data being the same texts with all punctuation, spaces, and footnote/endnote markers removed. In our study, we process each book individually. Each book is loaded into IDyOT, the lowest representation level being letters, and the resulting IDyOT sequential memory is then examined to evaluate the contribution of the successive layers.

Our evaluation measure is the percentage of words that are correctly found in their individually correct position in the text; that is, we are counting tokens and not types, and the position of the word in the text matters, so each occurrence of, for example, “the” is counted separately, and each occurrence must be individually correct to be counted. Thus, we use a stringent measure. To assess the impact of the hierarchy, we also record the lowest level at which each word is found. Position is important because, in due course, we will want to study the dynamic process of incremental learning that is recorded in IDyOT memory. From this perspective, there are 36,128, 219,986, and 127,368 words in the three datasets, one for each book, respectively. By way of baseline, we have computed two different empirical chance outcomes, and to evaluate the contribution of the re-representation in this case, we compare the number of words found cumulatively at each successive layer.

#### 6.2.2. Results

The results of the study are shown in Table 1.

**Table 1:** Results of the preliminary study.

**Dataset**	**The Time Machine**		**Moby Dick**		**Pride and Prejudice**	
**Words**	36,128		219,986		127,368	
**Baseline A**	0.09%		0.07%		0.06%	
**Baseline B**	0.11%		0.08%		0.08%	
**Δ**	**§6.2.2**	**§4.4**	**§6.2.2**	**§4.4**	**§6.2.2**	**§4.4**
α = δ = 0	6,416 (17.8)	5,885 (16.3)	36,105 (16.4)	31,601 (14.4)	24,459 (16.6)	20,171 (14.3)
α = δ = 1	4,377 (12.1)	4,133 (11.7)	29,938 (13.6)	25,821 (11.7)	20,003 (13.6)	18,497 (13.1)
α = δ = 2	1,018 (0.1)	1,804 (5.3)	8,149 (3.7)	15,348 (7.0)	5,243 (3.6)	8,947 (6.3)
α = δ = 3	44 (0.0)	526 (1.6)	336 (0.2)	4,539 (2.1)	270 (0.2)	2,079 (1.5)
α = δ = 4	0 (0.0)	52 (0.1)	4 (0.0)	556 (0.3)	1 (0.0)	401 (0.0)
α = δ = 5	0 (0.0)	4 (0.0)	0 (0.0)	45 (0.0)	0 (0.0)	27 (0.0)
α = δ = 6	0 (0.0)	0 (0.0)	0 (0.0)	1 (0.0)	0 (0.0)	1 (0.0)
Total	11,855 (32.8)	12,652 (35.0)	74,532 (33.9)	77,911 (35.4)	49,976 (39.2)	50,123 (39.6)

Our chance baseline comparisons were computed as follows. In Baseline A, we marked *n* − 1 boundaries at random positions in each dataset. We then evaluated the segmentation result as above, repeating the process and taking the mean of 100 such random segmentations. In Baseline B, we calculated the observed likelihood of a boundary at each position, assuming a uniform distribution. We then counted sequentially through each dataset, marking each possible boundary as a boundary with that probability. We then evaluated the result as above, repeating the process and taking the mean of 100 such random segmentations. The baselines are shown, with the rest of the results, in Table 1. It is immediately clear that our results are substantially better than either kind of chance.

Each row in the table shows the result of the application of a bigram model for segmentation using two different boundary detector functions, for each dataset. Each row shows the number of words correctly identified for the first time at that level. Two different boundary detection functions are shown: a simple increase in *h*, thus, labeled after this section of the paper:

$$\Delta_{\tau }^{\delta ,\alpha }=\{\begin{array}{cc} \text{true} & \text{if}h(\delta ,\alpha ,\tau )<h(\delta ,\alpha ,\tau_{+1}),\\ \text{false} & \text{otherwise.} \end{array}$$

and the more complex Δ function defined in section 4.4, and so labeled. The purpose of this comparison was exploratory: Occam's razor suggests that a simpler function would be preferable, so it is appropriate to consider various possibilities. More will be considered in future work.

We have shown the results for each layer of the hierarchy in which words are found (none are found beyond level 6), and for the total for each dataset. Figures in round brackets are percentage success, in the terms described above.

**Figure 7:**
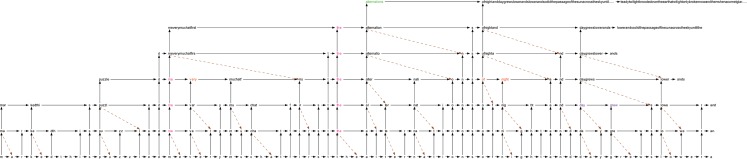
A visualization of actual IDyOT memory for the text fragment “…marked. This puzzled me very much at first. The alternations of night and day grew slower…,” taken at random from The Time Machine. For clarity, we have omitted higher levels at the beginning of the example, because they carry forward information from earlier data, that is not included in the example text.

Figure 7 shows a fragment of the IDyOT internal representation, in the same form as illustrated throughout this paper, for the text fragment “…marked. This puzzled me very much at first. The alternations of night and day grew slower…”, taken at random from The Time Machine. For clarity, we have omitted higher levels at the beginning of the example, because they carry forward information from earlier data, that is not included in the example text.

#### 6.2.3. Discussion

At the gross level, the first striking result is that the addition of the simplest version of re-representation in IDyOT results in an increase in successfully detected words by a factor of two or more. This is a substantial improvement, and it should be noted that it is achieved without any extra machinery (and in particular without *ad hoc* machinery): the Markov models are simply built in the usual way, one on top of the other, incrementally, as described above. While the absolute numbers do not compete with other systems on word-identification tasks, that is not the point here: the point is to demonstrate that re-representation, even in this simple way, improves the predictive power of the model.

A second interesting result is that the complex Δ, though performing better than the simple one overall, does less well at the lower levels. We speculate that this may lead us to use the simpler function once consolidation is implemented.

Thirdly, it is interesting to note how uniform is the performance of both the basic Markovian idea, but also of its hierarchical extension in IDyOT, over our three datasets.

In all, this small demonstration serves to show that there is promise in the IDyOT theory, though significantly more work is necessary before strong claims can be made.

## 7. Conclusion: Creativity, Representation and Consolidation in the Information Dynamics of Thinking

We now return to the central point of the current paper: that both incremental learning and consolidation should be seen as a process of repeated re-representation of an organism's experience of the world, whether the organism is natural or artificial. Put another way: learning is a process of meaning-making, which is performed by building representations, using representational affordances, whose propriety in local context is inferred from the structure of the data as perceived. Such meaning-making is incremental in time, because an organism needs to respond in real time to the world (in order to survive). However, the resulting memory model can be optimized, in terms of its predictive power (and thus in terms of its utility to the organism), by means of consolidation. Consolidation, in turn, may adapt the semantic spaces and/or the resulting segmentations. IDyOT's information-efficiency criterion affords a heuristic to guide such operations, which is independent of the domain of data. It is therefore capable of unsupervised, or implicit, learning, as are humans and animals.

The learning process formalized here encompasses both segmentation (chunking) in time and the construction of semantic representations. A fundamental point of the model is that, when a new set of representational affordances (i.e., a new semantic space) is constructed, the spaces from which it was constructed are not discarded, but persist, permitting the organism multiple different ways of conceptualizing its world, simultaneously. Furthermore, multiple spaces can be generated at any level of abstraction from any given stimulus, affording the possibility of multiple views of the same data, as is self-evident in human perception of speech and music. Conventionally, these multiple dimensions are considered as given; IDyOT theory proposes an account of how they are generated.

A corollary of the proposal above is that the representational affordances of each semantic space must themselves be constructed. Therefore, hypothesize, along with (Kemp and Tenenbaum, 2008), that there should be atomic building-blocks and construction operations for such spaces, which, in biological organisms, are presumably the basic cognitive operations of the wetware. The current paper goes a modest way to suggesting the mathematical nature of these cognitive tools: future work will investigate further.

There are multiple points where IDyOT memory construction operations offer the opportunity for creativity, interpreted again as incubation and inspiration (Wallas, 1926). The most important is the construction of concepts. This operation is often presented as inference in the machine learning literature, but to do so assumes that meaning exists in the world as a ground truth, and this author does not subscribe to this view. Rather, concept formation (the identification of a coherent concept by a natural or an artificial cognitive entity) consists in the construction of a semantic space in which dimensions and geometry coincide to give it coherent meaning ^15^. Another opportunity for creativity is IDyOT's capacity to move points from one category to another within a space (Wiggins, 2018). This connotes the kind of creativity in which an existing piece of knowledge is viewed from a new perspective. Finally, the capacity for re-representation within the process of learning affords creativity in its own right, not only of concepts (as above) but of semantic/conceptual spaces themselves (Gärdenfors, 2000). Because such spaces are geometrical, they can be explored: new points can be interpolated between, or extrapolated beyond, existing points, and thus can imagination and creativity be given a palette from which to draw their metaphorical colors.

The kind of creativity that drives language and music (on both everyday and world-changing levels) can perhaps be explained by the sequential and predictive elements of IDyOT, and a particularly unusual feature of the theory is the ability to reason abstractly, at levels that represent multiple possible instantiations of, perhaps, a sentence or melody. This is an important feature of human reasoning, which is rarely addressed in AI research—a notable exception being the “middle-out reasoning” of Bundy et al. (2005). We contend that this an important aspect of human creativity that needs to be reflected in autonomous artificial creative systems of the future.

## Author Contributions

The model described here is the work of GW, though it has been inspired by many sources (see Acknowledgments, below), most notably the work of Conklin and Pearce, which are cited in the paper. The implementation and studies described here are the work of AS. The writing is entirely the work of GW.

### Conflict of Interest Statement

The authors declare that the research was conducted in the absence of any commercial or financial relationships that could be construed as a potential conflict of interest.

## Footnotes

^1^Wiggins (2011) defines a descriptive model of a cognitive process as one which predicts its input-output relation correctly, but which makes no claim to explicate its mechanism. On the other hand, an explanatory model does claim to explicate mechanism, at a level of abstraction which is less than that of a corresponding descriptive model, though not necessarily in full detail.

^2^Information Dynamics of Music.

^3^Wiggins (2011) describes a research methodology based on the alternation of descriptive and explanatory cognitive models as one moves from very high level descriptions of effects (e.g., the Gestalt principles) to detailed explanations of the operation of neurons (as in the Human Brain Project https://www.humanbrainproject.eu/), by progressive, incremental steps of understanding, each at a different level of abstraction.

^4^This problem will be a future target for IDyOT research.

^5^The notation τ_+*i*_ is used instead of the more obvious τ + 1 because it is not always the case that τ_+*i*_ = τ + *i*—see Figure 3.

^6^From an implementation perspective, this can be implemented efficiently by updating a matrix incrementally as input is processed.

^7^This capacity is analogous to the viewpoints of Conklin and Witten (1995) of the structure in each level.

^8^See http://ismir.net.

^9^Other solutions exist: for example, dolphins sleep with just half of their brain at any one time.

^10^Terminology: Chella et al. (2008) refer to knoxels, minimal regions in conceptual spaces. Here, the symbols of any *A*^δ,α^ are the same thing.

^11^This is very clearly not a Chomskian grammar, but something more based in semantics, and learned bottom up.

^12^This latter is a percept that is learned by some musicians; it is, regrettably, inexplicable to those who have not learned it.

^13^Specifically: it is familiar with the other words in the sentence with enough context to recognize them; and it has identified “s” as an ending.

^14^https://www.gutenberg.org

^15^Perhaps this would translate to there being physical properties of a biological neural substrate which support the oscillatory behavior that is modeled by IDyOT's Hilbert spaces?
